# Willingness to pay for a group and an individual version of the Lifestyle-integrated Functional Exercise program from a participant perspective

**DOI:** 10.1186/s12889-022-14322-2

**Published:** 2022-10-18

**Authors:** Sophie Gottschalk, Hans-Helmut König, Michael Schwenk, Corinna Nerz, Clemens Becker, Jochen Klenk, Carl-Philipp Jansen, Judith Dams

**Affiliations:** 1 Department of Health Economics and Health Services Research, Hamburg Center for Health Economics, University Medical Center Hamburg-Eppendorf, Hamburg, Germany; 2 Network Aging Research, Heidelberg University, Heidelberg, Germany; 3 Department of Clinical Gerontology and Geriatric Rehabilitation, Robert Bosch Hospital, Stuttgart, Germany; 4 Institute of Epidemiology and Medical Biometry, Ulm University, Ulm, Germany; 5 IB University of Applied Health and Social Sciences, Study Centre Stuttgart, Stuttgart, Germany; 6 Human Performance Research Centre, Department of Sport Science, University of Konstanz, Konstanz, Germany; 7 Institute of Sports and Sports Sciences, Heidelberg University, Heidelberg, Germany

**Keywords:** Willingness to pay, Patient preferences, Participant perspective, Contingent valuation method, Fall prevention, Physical activity promotion

## Abstract

**Background:**

Perceived benefits of intervention programs from a participant perspective can be examined by assessing their willingness to pay (WTP). Aiming to support decision-makers in their decision to implement a fall prevention program, this study examined (1) the WTP for a group-based and an individually delivered fall prevention program, (2) which factors influence WTP, and (3) whether the WTP exceeds the intervention costs.

**Methods:**

WTP was elicited using Payment Cards from 237 individuals who participated in a randomized non-inferiority trial (LiFE-is-LiFE) comparing a group version of the Lifestyle-integrated Functional Exercise program (gLiFE) with the individually delivered version (LiFE). Linear regression models were used to examine factors associated with WTP. The net benefit for (g)LiFE was calculated as the difference between WTP and intervention costs, assuming different scenarios of intervention costs (varying group sizes of gLiFE) and hypothetical subsidy levels by a payer (€0, €50, or €75).

**Results:**

The mean WTP was €196 (95% CI [172, 221]) for gLiFE and €228 (95% CI [204, 251]) for LiFE. In the linear regression model, WTP was significantly associated with delivery format (−€32, 95% CI [− 65, − 0.2], for gLiFE) and net household income (+ 68€, 95% CI [23, 113], for ≥€3000 compared to <€2000). The net benefit for gLiFE was positive in most cases. Due to higher intervention costs of LiFE compared to gLiFE (€298 vs. €113), the net benefit for LiFE was negative for the majority of the sample, even at a subsidy of €75.

**Conclusion:**

The results provide insight into how valuable the interventions are perceived by the participants and thereby may be used by decision-makers as complement to cost-effectiveness analyses. WTP for both programs was generally high, probably indicating that participants perceived the intervention as quite valuable. However, further research is needed on the WTP and net benefit of fall prevention programs, as results relied on the specific context of the LiFE-is-LiFE trial.

**Supplementary Information:**

The online version contains supplementary material available at 10.1186/s12889-022-14322-2.

## Introduction

In the context of demographic change, the development of effective intervention programs to promote health into old age has become a priority in societies with an increasing population of older people. Besides clinical effectiveness, the (widespread) implementation of a program also depends on available resources, on whether the benefits of the program outweigh the costs associated with the implementation, and on who bears the costs of the program. Depending on the perspective, different benefits and costs are of relevance when deciding in favor of or against the implementation of a program. In economic analyses of healthcare programs, a societal or payer’s perspective is frequently adopted, assuming that intervention costs are (at least partly) covered by the state or a health insurer [1]. However, health and well-being are also perceived as individual responsibility and hence can be seen as a good that people are willing to invest into. According to welfare economic theory, the benefits of a good or service are reflected in the form of willingness to pay (WTP) – the maximum amount of money an individual is willing to give up for the good or service. Thus, WTP is a concept that can be used to assign a monetary value to a good. Thereby, WTP goes beyond health and, unlike, for example, quality-adjusted life years (QALYs), which are frequently being used as effect measure in economic evaluations, does not restrict participants to express their preferences on pre-specified dimensions [2, 3]. Knowing the strength of a preference, aka how much people would be willing to pay, could also be relevant from a payer’s perspective who may opt for a cost subsidy rather than full coverage of an intervention.

WTP for healthcare interventions is frequently captured using stated preference methods, which can be classified into direct (e.g., payment cards) and indirect (e.g., discrete choice experiments) methods [4]. In indirect methods, individuals are typically presented with several intervention options that differ in, e.g., the intervention characteristics, expected effects, and price. Participants are then asked to choose their preferred intervention option. In direct methods, on the other hand, individuals’ WTP is determined by directly asking how much individuals would be willing to pay for an intervention. When collecting WTP data from participants of a clinical trial after completing the intervention, the stated WTP is assumed to reflect their individual perception of benefits, which may go beyond clinically visible effects such as improved physical performance or QALYs.

In Germany, the promotion of healthy ageing has been defined as national health goal [5]. Expanding measures to prevent falls is defined as sub goal since falls have a high prevalence with around one third of the population aged 65 years and older experiencing a fall at least once per year [6–8]. Falls can lead to injuries (e.g., hip fractures) which have serious consequences on health, quality of life and the healthcare budget [9]. Effective fall prevention programs could therefore be of high relevance for the promotion of healthy ageing and reducing the economic burden of falls. The LiFE-is-LiFE project compared a group-delivered version of the Lifestyle-integrated Functional Exercise program (gLiFE) with the original, individually delivered version (LiFE) [10]. Both programs consist of strength and balance activities that are integrated into everyday routines. In both programs, falls were reduced and physical activity was improved, while gLiFE was less costly in terms of intervention costs [11, 12]. Moreover, a content evaluation showed that both program versions were similar in terms of perceived safety, intensity of the exercises, integrability, and acceptance [13]. To our knowledge, no study has assessed how much participants are willing to pay for an exercise program aiming to maintain physical function and activity and reduce the risk of falling. When it comes to individual preferences and perceived benefits beyond clinical effectiveness, one could assume that the WTP of such a program differs by mode of administration. For example, gLiFE may be perceived as more valuable since it involves a social component (e.g., increased motivation through peer support) or, on the other hand, LiFE might be preferred as the individual training in the participant’s home may be perceived as an advantage for implementing the LiFE activities into daily routines [13, 14].

Therefore, the aim of the current study was to explore WTP for gLiFE and LiFE, to examine factors influencing WTP, and to examine whether the perceived benefits – operationalized as WTP – exceed the costs associated with conducting the intervention(s).

## Methods

### Study design and sample

Data was taken from the LiFE-is-LiFE study (registered on 12/03/2018 under clinicaltrials.gov, identifier: NCT03462654), a multi-center, two armed, single-blinded, randomized non-inferiority trial, including community-dwelling, German-speaking people aged ≥ 70 years at risk of falling, who were able to ambulate 200 m without personal assistance [10]. Participants were randomized to either LiFE or gLiFE. Data was obtained at three time points (baseline, 6 months, and 12 months). WTP was assessed at 12 months.

### Interventions and intervention costs

LiFE consisted of seven home visits (≈ 1 h) where a trainer presented activities for balance, strength, and general physical activity, adapting the performance and uptake of the activities to the needs of the participants. The trainer gave instructions on how to independently execute these activities and helped in implementing these activities in an individual participant’s daily routine. In gLiFE, the program was taught by two trainers in seven sessions (≈ 2 h) to groups of 8 to 12 participants. The intervention sessions followed a detailed curriculum as trainers were not able to adapt flexibly to each individual’s preferences. In both intervention arms, the participants received 2 additional ‘booster phone calls’ 4 and 10 weeks after the last intervention session. A detailed description of the interventions (including a TIDieR checklist) can be found in the study protocol [10]. The development of the conceptual gLiFE framework and a content analysis as well as a qualitative analysis of the acceptance of the two program versions were published separately [13–15].

Intervention costs for gLiFE and LiFE which incurred for the training sessions and booster phone calls were calculated as costs per participant based on personnel and material costs and travel expenses, assuming group sizes of 12 (scenario 1, base case), 10 (scenario 2), or 8 participants (scenario 3) in gLiFE. Assumptions underlying the calculation of different scenarios are presented in Table A1 (Additional file 1). For each scenario, the amount of costs from the participant perspective was derived by subtracting different hypothetical levels of subsidy (e.g., by a health insurer) of €0, €50, and €75.

### Willingness to pay

Participants’ WTP was elicited using Payment Cards, which are commonly used for assessing WTP for healthcare interventions [16]. Using response categories from €0, €5, €10, €20 to ‘more than €100’, participants receiving LiFE or gLiFE were asked about the amount of money they would surely be willing to pay as well as the amount they would definitely not be willing to pay for one training session of the respective program. The WTP for one training session was determined as the mean between these two values, which was then multiplied by the number of training sessions to obtain the total WTP for the intervention.

### Explanatory variables

The following sample characteristics were considered in the analyses: intervention group (gLiFE/LiFE), age, sex, marital status, net household income, health insurance status (statutory vs. private), number of chronic conditions, healthcare costs, baseline fall status (non-faller vs. faller in the previous 6 months), motivation to exercise, satisfaction with the program, and training frequency (number of LiFE activities performed per week) at 12-month follow-up.

For the calculation of healthcare costs, costs from inpatient and outpatient service utilization, as well as medication and formal care use in the previous 6 months before the baseline assessment were considered. Resource utilization was monetarily valued in Euro (€) based on standardized unit costs [17] and inflated to the year 2018 [18].

Motivation to exercise was measured based on the autonomous motivation score of the Behavioral Regulation in Exercise Questionnaire (BREQ-3) [19], ranging from 0 to 4, with higher scores indicating higher motivation.

Satisfaction with the program was measured on a 5-point Likert scale (higher scores indicate higher satisfaction) and by a German school grade system using response categories from 1 (best grade) to 6 (worst grade).

### Statistical analysis

The WTP was descriptively analyzed for persons with different sample characteristics for the total sample as well as for gLiFE and LiFE separately. Potential determinants of WTP were examined by linear regression models including the group variable (gLiFE/LiFE), sex, age, income, number of chronic conditions, healthcare costs, and motivation to exercise as independent variables. The mean net benefit from the participant perspective was calculated for different intervention scenarios (varying group sizes in gLiFE) and levels of subsidy by subtracting intervention costs from the WTP. The incremental net benefit of gLiFE over LiFE was determined by linear regression models adjusted for the potential determinants mentioned above.

Skewness of data was taken into account using a bootstrapped sample with n = 1,000 replicates. All analyses were conducted using STATA/SE 16.0 [StataCorp. 2019. Stata Statistical Software: Release 16. College Station, TX: StataCorp LLC]. The significance level was set to 0.05.

## Results

### Sample characteristics

Sample characteristics are displayed in Table 1. Two hundred and thirty seven participants of the LiFE-is-LiFE trial completed the payment card at 12-month follow-up. Of those, the majority were female (74%), had an intermediate or high education (67%), were married/living in a partnership (45%) or widowed (37%), and insured by statutory health insurance (74%). The mean age was 79 years and 41% had fallen at least once in the previous 6 months before the baseline assessment. On average, participants performed 53 LiFE activities per week and were overall satisfied with the program (mean satisfaction = 4.7 [maximum 5]; mean “school grade” = 1.6). Sample characteristics were similar for gLiFE and LiFE and between drop-outs and completers (results not shown).

**Table 1:** Sample characteristics

		Total (n = 237)			gLiFE (n = 117)			LiFE (n = 120)	
**Female**	n (%)	176	(74.26)		87	(74.36)		89	(74.17)
**Age**	mean (SE)	78.66	(0.36)		78.52	(0.52)		78.80	(0.49)
**Educational degree** ^**1**^	n (%)								
low		71	(29.96)		33	(28.21)		38	(31.67)
intermediate		67	(28.27)		31	(26.50)		36	(30.00)
high		93	(39.24)		48	(41.03)		45	(37.50)
other/no degree		6	(2.53)		5	(4.27)		1	(0.83)
**Marital status**	n (%)								
married/living in a partnership		107	(45.15)		57	(48.72)		50	(41.67)
widowed		87	(36.71)		38	(32.48)		49	(40.83)
divorced		28	(11.81)		17	(14.53)		11	(9.17)
permanently living separated		3	(1.27)		1	(0.85)		2	(1.67)
single		12	(5.06)		4	(3.42)		8	(6.67)
**Net household income**	n (%)								
€500 to <€750		5	(1.98)		2	(2.14)		2	(1.83)
€750 to <€1000		5	(2.24)		2	(1.79)		3	(2.67)
€1000 to <€1500		27	(11.27)		15	(12.65)		12	(9.92)
€1500 to <€2000		52	(22.03)		22	(18.89)		30	(25.08)
€2000 to <€3000		70	(29.45)		33	(28.55)		36	(30.33)
€3000 to <€5000		58	(24.51)		32	(27.01)		26	(22.08)
€5000+		20	(8.52)		11	(8.97)		10	(8.08)
**Health insurance status**	n (%)								
statutory		175	(73.84)		82	(70.09)		93	(77.50)
private		62	(26.16)		35	(29.91)		27	(22.50)
**Number of chronic conditions**	mean (SE)	2.44	(0.10)		2.43	(0.14)		2.45	(0.13)
**Healthcare costs in €**	mean (SE)	1585.60	(171.03)		1375.36	(179.37)		1790.57	(288.57)
**Prevalence of fallers**	n (%)	98	(41.35)		52	(44.44)		46	(38.33)
**Number of falls among fallers**	mean (SE)	1.65	(0.13)		1.62	(0.20)		1.70	(0.18)
**Motivation to exercise** (range 0–4)^**2,3**^	mean (SE)	2.96	(0.05)		3.00	(0.07)		2.92	(0.08)
**Satisfaction with the program** (max. = 5)^**2**^	mean (SE)	4.69	(0.06)		4.61	(0.08)		4.77	(0.08)
**“School grade"** ^**4**^	mean (SE)	1.60	(0.04)		1.62	(0.06)		1.58	(0.05)
**Training frequency** ^**5**^	mean (SE)	53.46	(1.50)		53.20	(2.26)		53.71	(2.00)

^1^ low (9 years of school education), intermediate (10 years of school education), high (qualifies to enter university)

^2^ higher scores indicate higher motivation/satisfaction

^3^ BREQ-3 autonomous motivation score at FU12

^4^ „school grade“: 1 (A) =“sehr gut“, 2 (B) = “gut“, 3 (C) =“befriedigend“, 4 (D) = “ausreichend“, 5 (E) = „mangelhaft“, 6 (F)= „ungenügend“

^5^ training frequency = number of LiFE activities performed per week

### Willingness to pay

The mean WTP stratified by groups of sample characteristics is displayed in Table 2. In the total sample, gLiFE participants had a lower mean WTP than LiFE participants (€196, 95% CI [172, 221] vs. €228, 95% CI [204, 251]) and participants with an income of €2000 to <€3000 or ≥€3000 had a higher mean WTP than those with an income of €500 to <€2000 (€218, 95% CI [187, 248] / €250, 95% CI [215, 284] vs. €175, 95% CI [152, 199]). Moreover, WTP was higher in males (€245, 95% CI [207, 283] vs. €201, 95% CI [183, 219], privately insured participants (€249, 95% CI [213, 285] vs. €200, 95% CI [181, 218]), those with higher healthcare costs (tertile 3: €235 95% CI [202, 268]; tertile 1: €185, 95% CI [156, 215]), and those with lower motivation to exercise (score ≤ 3: €238, 95% CI [210, 265]; score > 3: €190, 95% CI [169, 210]).

**Table 2:** Older adults’ willingness to pay (€) for the gLiFE/LiFE intervention by sample characteristics

	Total (n = 237)			gLiFE (n = 117)			LiFE (n = 120)	
	Mean	(95% CI)		Mean	(95% CI)		Mean	(95% CI)
**Group**								
gLiFE	196	(172, 221)						
LiFE	228	(204, 251)						
**Age**								
< 80	219	(198, 241)		208	(175, 241)		230	(200, 259)
80+	202	(176, 228)		180	(143, 217)		225	(188, 262)
**Sex**								
Male	245	(207, 283)		226	(171, 280)		264	(211, 316)
female	201	(183, 219)		186	(160, 213)		216	(191, 240)
**Marital status**								
married/living in partnership	224	(197, 251)		211	(172, 250)		238	(197, 280)
widowed	197	(172, 222)		161	(131, 191)		225	(188, 262)
divorced	231	(181, 280)		248	(172, 325)		204	(171, 236)
permanently living separated	257	(165, 349)		175	(175, 175)		298	(218, 377)
single	169	(108, 231)		109	(43, 176)		199	(112, 286)
**Net household income**								
**€**500 to <**€**2000	175	(152, 199)		160	(124, 196)		189	(159, 219)
**€**2000 to <**€**3000	218	(187, 248)		203	(162, 245)		231	(189, 273)
**€**3000+	250	(215, 284)		227	(181, 273)		276	(226, 326)
**Health insurance status**								
statutory	200	(181, 218)		184	(156, 212)		213	(189, 238)
private	249	(213, 285)		226	(178, 273)		279	(224, 333)
**Number of chronic conditions**								
0–2 (ref.)	212	(190, 235)		194	(162, 226)		233	(202, 263)
3	195	(154, 235)		151	(95, 207)		217	(167, 266)
4–7	229	(195, 263)		228	(184, 271)		231	(180, 281)
**Healthcare costs**								
tertile 1 (≤€570)	185	(156, 215)		168	(120, 216)		203	(166, 239)
tertile 2 (>€570 to ≤€1,132)	217	(193, 241)		199	(173, 225)		234	(198, 271)
tertile 3 (>€1,132)	235	(202, 268)		222	(174, 270)		247	(203, 292)
**Fall status**								
non-faller	209	(189, 229)		188	(161, 215)		228	(199, 256)
Faller	217	(188, 246)		207	(163, 250)		229	(190, 267)
**Motivation to excercise** ^1^								
lower medium (score ≤ 3)	238	(210, 265)		222	(183, 260)		252	(215, 288)
upper medium (score > 3)	190	(169, 210)		176	(145, 206)		205	(178, 232)
**Satisfaction with the program**								
(rather) unsatisfied	208	(145, 270)		159	(73, 246)		263	(180, 345)
rather satisfied	223	(188, 258)		212	(164, 260)		235	(188, 282)
(very) satisfied	207	(187, 227)		193	(163, 223)		220	(195, 246)
**“School grade"** ^2^								
D/C (ref.)	201	(131, 271)		165	(111, 218)		273	(105, 441)
B	217	(192, 242)		209	(170, 247)		224	(192, 256)
A	209	(184, 233)		191	(154, 228)		227	(196, 258)
**Training frequency** ^3^								
lower 3rd (0–42)	198	(170, 225)		197	(159, 235)		198	(162, 235)
middle 3rd (43–63)	217	(189, 245)		190	(142, 239)		237	(203, 271)
upper 3rd (64–112)	221	(189, 254)		201	(157, 244)		245	(198, 291)

^1^BREQ-3 autonomous motivation score at FU12

^2^„school grade“: 1 (A) =“sehr gut“, 2 (B) = “gut“, 3 (C) =“befriedigend“, 4 (D) = “ausreichend“, 5 (E) = „mangelhaft“ (not reported), 6 (F)= „ungenügend“ (not reported)

^3^number of LiFE activities performed per week

In the linear regression model identifying the determinants of WTP (Table 3), gLiFE was associated with a significantly lower WTP (−€32, 95% CI [− 65, − 0.2]) compared to LiFE. Among the other potential determinants in the model, only income was significantly associated with WTP, with the highest income group having a €68 (95% CI [23, 113]) higher WTP compared to the lowest income group.

**Table 3:** Determinants of older adults’ willingness to pay for the (g)LiFE intervention

	Beta	SE	95% CI	p-value
gLiFE (ref. LiFE)	−32	16	(− 64.61, − 0.16)	0.049
Female (ref. male)	−19	23	(− 63.33, 25.73)	0.408
Age	3	2	(− 0.16, 5.98)	0.063
Net household income (ref. <€2000)				
€2000-€3000	32	20	(− 8.02, 72.23)	0.117
€3000+	68	23	(23.21, 112.96)	0.003
Number of chronic conditions	2	5	(− 8.78, 12.69)	0.721
Healthcare costs	0	0	(− 0.01, 0.01)	0.727
Motivation to exercise^2^	−22	11	(− 44.31, 0.63)	0.057
Intercept	40	121	(− 196.46, 275.91)	0.742
*Adjusted R-Squared*	*0.071*			

^2^ BREQ-3 autonomous motivation score at FU12

### Net benefit

The intervention costs per participant for LiFE were €298. For gLiFE, intervention costs varied depending on the group size with €113, €123, and €138 for 12, 10, and 8 participants, respectively (Table A1, Additional file 1). In the base case scenario, gLiFE had a significant positive mean net benefit between €83 (95% CI, [59, 107]) at €0 subsidy and €158 (95% CI, [134, 182]) at €75 subsidy (Table 4). When lower group sizes were assumed (Scenario 2 and 3), the mean net benefit was somewhat lower, but remained positive for each subsidy level. For LiFE, the intervention costs exceeded the WTP, resulting into negative mean net benefits, except for the case that €75 were subsidized (€5, 95% CI [− 19, 28]).

**Table 4:** Mean net benefit by intervention groups, scenarios of intervention costs, and subsidy schemes

			gLiFE		LiFE		Difference	
	Subsidy		Mean	(95% CI)	Mean	(95% CI)	Mean	(95% CI)
**Scenario 1**	€0		83	(59, 107)	−70	(−94, −47)	153	(119, 188)
	€50		133	(109, 157)	−20	(−44, 3)		
	€75		158	(134, 182)	5	(−19, 28)		
**Scenario 2**	€0		73	(49, 98)	-70	(−94, −47)	143	(109, 178)
	€50		123	(99, 148)	-20	(−44, 3)		
	€75		148	(124, 173)	5	(−19, 28)		
**Scenario 3**	€0		58	(34, 83)	-70	(−94, −47)	129	(94, 163)
	€50		108	(84, 133)	-20	(−44, 3)		
	€75		133	(109, 158)	5	(−19, 28)		

**Notes**: Scenarios 1–3 differ by group size for gLiFE which influenced the intervention costs: scenario 1 (base case, 12 participants, €113), scenario 2 (10 participants, €123), scenario 3 (8 participants, €138). Intervention costs for LiFE were €298.

**Fig. 1:**
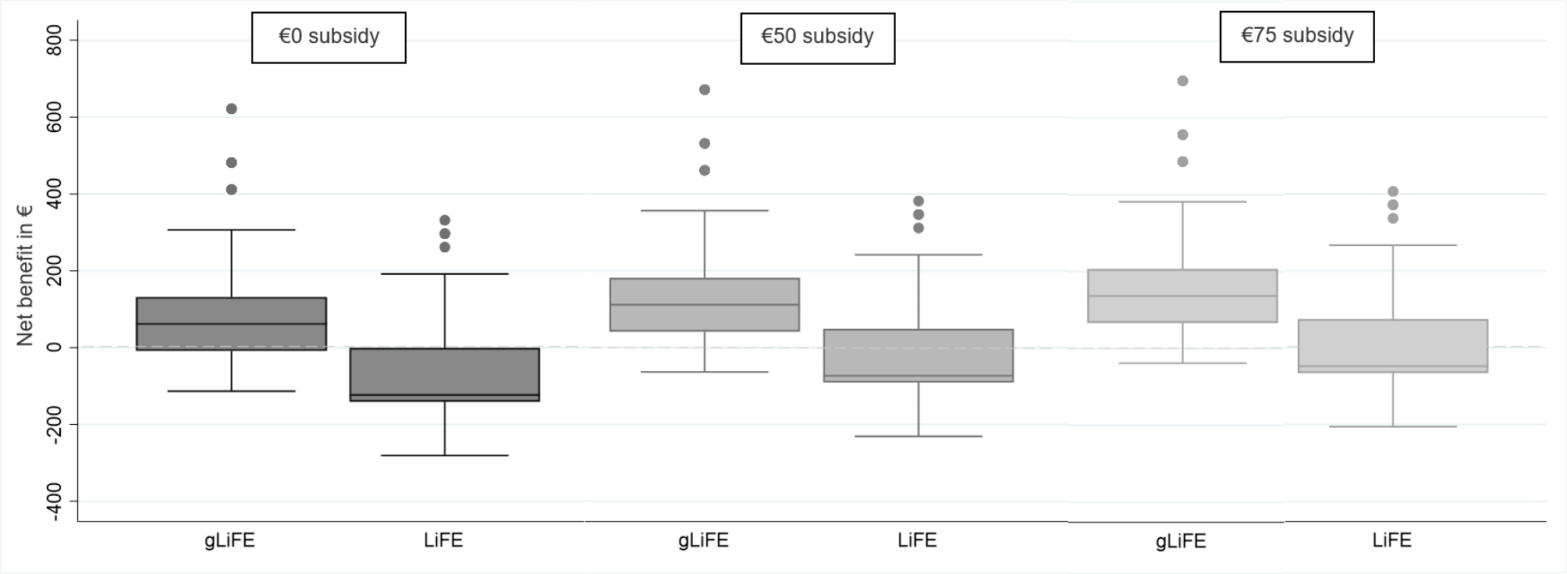
Distribution of the mean net benefit for gLiFE/LiFE by different subsidy levels of intervention costs. Intervention costs based on Scenario 1 (gLiFE: €113; LiFE: €298)

When the distributions of the net benefit for gLiFE and LiFE were graphically examined for scenario 1 by different subsidy levels (Fig. 1), it could be observed that the majority of gLiFE participants (68% [€0 subsidy], 86% [€50 subsidy], and 95% [€75 subsidy]) had a positive net benefit, whereas this applied to only 25% (€0 subsidy), 29% (€50 subsidy), and 40% (€75 subsidy) of LiFE participants

The unadjusted incremental net benefit for gLiFE compared to LiFE was €153 (95% CI [119, 188],Table 4). Adjusting the incremental net benefit did not change the estimate relevantly (€154, 95% CI [122, 186]; not displayed in table)

## Discussion

This study is the first that assessed the WTP for a group-based and an individually delivered version of a fall prevention and activity promotion program (LiFE) in a sample of community-dwelling German older adults at risk of falling. WTP for both programs was generally high, probably indicating that participants perceived the intervention as quite valuable and thus possibly reflecting a demand for such interventions. WTP was determined by delivery format and income, with LiFE participants on average reporting €32 higher WTP than gLiFE participants and higher income groups reporting higher WTP. For gLiFE, benefits in terms of WTP exceeded intervention costs in most cases, while LiFE had considerably higher intervention costs than gLiFE (€298 vs. €113), and thus the WTP was lower than the intervention costs in the majority of the sample (60–75%, depending on hypothetical subsidy level). Hence, the difference in WTP between gLiFE and LiFE did not compensate for the higher intervention costs (+€185), even when subsidized by up to €75.

Asking participants of an intervention study who have actual experience with the intervention of interest about their willingness to pay (rather than reporting their WTP for hypothetical intervention scenarios) has not been done frequently, especially in the field of physical activity interventions for community-dwelling older people. However, this approach might be an attractive complement to the evaluation of (cost-)effectiveness of competing interventions – WTP constitutes a measure of the perceived benefits or value of the intervention from a participant perspective that is not restricted to predefined dimensions on which benefits can be expressed (e.g., in patient-reported outcome measures). As WTP may be based on factors other than effectiveness alone [20], knowing the preferences (WTP) of the target population may be particularly useful when the effectiveness of different program formats based on conventional measures (e.g. reduction of falls [11, 12]) is indifferent or similar. Furthermore, WTP extends conventional (cost-)effectiveness frameworks, as it may reveal additional benefits perceived by the participants that may otherwise have been overlooked or not captured. For example, in RCTs that evaluated exercise interventions for older people, only marginal (and probably not clinically important) differences in QALYs between the intervention and control group are found, at least over time horizons of six months to two years [21]. Overall, determining the WTP may aid decision-makers in deciding which intervention should be preferred for implementation [2].

Beyond the level of willingness to pay, it is also interesting to know which factors influence willingness to pay, as this information may then be used to adapt interventions according to the preferences of the target group. In the current study, only income (besides program version) determined WTP, and overall only 7% of the variance was explained by the potential determinants in the multivariate regression model, indicating that other (unobserved) factors determine the WTP to a large extent. Other factors that can be hypothesized to determine WTP could be the individually perceived relevance (e.g. individually perceived risk of falling) and perceived effectiveness of the intervention, the presence of other health conditions whose treatment may be given a higher priority, or the relationship with the trainer [14]. It is also not clear whether participants factored the cost of providing the intervention, and thus the additional effort required for home visits in LiFE, into their willingness to pay, which may explain the difference in WTP between program versions [20].

That WTP is associated with income is not surprising as it is inherently limited by wealth [22]. This carries a danger of self-selection of only higher-income populations into participating in the program which poses a threat to the idea of equal health opportunities, for example, making prevention accessible to everyone independent of socio-economic position [23]. In Germany, prevention programs can be certified, which qualifies them for subsidies of the intervention costs by the health insurances. These subsidies lower the intervention costs and thereby make interventions more accessible to people that are economically less well of, while at the same time alleviating the burden on health insurers’ budgets. Assuming a subsidy of €75, the WTP of almost all gLiFE participants (95%) covered (or even exceeded) the intervention costs, providing a strong argument for the implementation of gLiFE over LiFE. However, it does not seem reasonable to give recommendations for not offering and/or subsidizing LiFE – there may be still demand for LiFE as between 25% and 40% of the LiFE participants were willing to pay enough to cover the intervention costs. Those people, based on individual preferences, may still opt for the individual program despite being more costly. For example, some people may prefer individual supervision and learning the program in their own home where the activities could be adapted to the individual conditions and are therefore more easy to integrate into everyday life, whereas for others the social aspects of a group program (e.g., motivation through peer support) may be more important [13, 14]. Moreover, the individual approach in LiFE could be more suitable for people for whom participation in group programs would be difficult, for example, because of physical and transport limitations.

Despite the reduction of access barriers through the subsidies, the uptake of the intervention still depends to a certain extent on the financial resources of the individual. This selection effect could be reduced if a payer fully reimburses the intervention costs, which may still be economically attractive for a payer if the effects of an intervention are expected to spill over to cost savings (e.g., lower health-related resource use). Therefore, assessing participants’ preferences in terms of their WTP does not substitute cost-effectiveness analyses, but may be used as a complement, especially when there are two competing interventions where neither is clearly superior to the other in terms of cost-effectiveness, as was the case with gLiFE and LiFE [12, 24].

### Limitations

Limitations arise from the fact that the elicited WTP is tied to the specific survey context and therefore does probably not reflect ‘real world’ behavior [22]. The results are based on a selective population of participants who can be assumed to have a special interest in fall prevention and may not be representative of the target population in terms of their socio-economic status, which influences WTP. Given that all participants valued their WTP based on their personal experience of the program, the WTP may not represent the WTP of individuals who do not have actual experiences with the program to draw on, and comparing the strength of the preference between gLiFE and LiFE based on this study may be limited since the participants were only asked about their WTP for the version they received (either gLiFE or LiFE). Moreover, the time point of assessing the WTP (at 12-month follow-up) could have influenced the valuation. Test-retest reliability of the WTP was not examined in this study and no information on the WTP of study drop-outs (n = 64) was available. Future studies may use a discrete choice experiment to find out which attributes determine the level of WTP or the decision for one or the other program version.

## Conclusion

This study explored WTP for a group-delivered and an individually delivered activity promotion and fall prevention program. The results are useful for decision-makers or potential payers as they provide insight into how valuable the interventions are perceived by the participants and thereby complement the cost-effectiveness studies of the LiFE program with a participant perspective. The high WTP for both programs suggests that they are perceived as valuable by the participants, thereby supporting previous results on the acceptability and content of the (g)LiFE program. WTP was associated with income and was higher for LiFE than for gLiFE, but this difference did not compensate the higher intervention costs in LiFE. gLiFE was likely to yield a positive net benefit, especially when the intervention is subsidized by a potential payer, while for the individual program the WTP was less likely to exceed the costs. Payers may still consider subsidizing both versions, as there may be a number of individuals who prefer the individual program despite its higher cost.

## Electronic supplementary material

Below is the link to the electronic supplementary material.

Supplementary Material 1

## Data Availability

The datasets generated and/or analyzed during the current study are not publicly available due to ethical and confidentiality concerns but are available from the corresponding author on reasonable request.

## List of abbreviations

**BREQ-3**: Behavioral Regulation in Exercise Questionnaire.
**gLiFE**: Group-based Lifestyle-integrated Functional Exercise.
**LiFE**: Lifestyle-integrated Functional Exercise.
**QALY**: Quality-adjusted life year.
**WTP**: Willingness to pay.
