# Supplementary material for: Willingness to pay for a group and an individual version of the Lifestyle-integrated Functional Exercise program from a participant perspective
Source: BMC Public Health. 2022 Oct 18;22:1934. doi: 10.1186/s12889-022-14322-2 (PMC9580107; doi:10.1186/s12889-022-14322-2)
Supplement: Supplementary file 1 — Supplementary Material 1 [file 12889_2022_14322_MOESM1_ESM.pdf]

Table A1 Data and assumptions for the calculation of intervention costs by scenario

**LiFE sessions**			
Average duration[1]		120 min	
**gLiFE sessions**			
Average duration[1]		150 min	
(Average) number of participants per group[2]		12 (scenario 1)	
		10 (scenario 2)	
		8 (scenario 3)	
Average travel expenses per participant		€ 17.92	
**Other assumptions**			
Salary level of the trainers		TVöD[3] E8	
Number of sessions per participant[2]		7	
Number and average duration of "booster phone calls"		2 x 30 min	
Costs material set for each participant		€ 28.65	
**Total intervention costs**	Scenario 1	Scenario 2	Scenario 3
gLiFE	€ 113.32	€ 123.26	€ 138.18
LiFE	€ 298.30	€ 298.30	€ 298.30

[1] including preparation and travelling time
[2] assumption: sessions have to be paid regardless of participation
[3] TVöD: civil service collective agreement; 2018 wages
